# Erucin inhibits osteoclast formation via suppressing cell–cell fusion molecule DC-STAMP without influencing mineralization by osteoblasts

**DOI:** 10.1186/s13104-022-05988-3

**Published:** 2022-03-16

**Authors:** Tomohiro Takagi, Hirofumi Inoue, Shungo Fujii, Nobuyuki Takahashi, Mariko Uehara

**Affiliations:** 1 Department of Nutritional Science and Food Safety, Faculty of Applied Bioscience, Tokyo University of Agriculture, 1-1-1 Sakuragaoka, Setagaya-ku, Tokyo, 156-8502 Japan; 2 Department of Nutritional Sciences, Faculty of Nutritional Sciences, Tohto University, 4-2-7, Nishi, Kamishiba-cyo, Fukaya-shi, Saitama, 366-0052 Japan; 3 Department of Health and Nutrition, Faculty of Human Science, Hokkaido Bunkyo University, 5-196-1, Koganechuo, Eniwa-shi, Hokkaido, 061-1449 Japan

**Keywords:** Erucin, Isothiocyanate, Osteoclast differentiation, Osteoclast cell fusion, DC-STAMP

## Abstract

**Objective:**

Erucin (ERN), an isothiocyanate, is derived from the vegetable arugula. Although ERN has antitumor and antioxidant activity, the effect of ERN on osteoclast and osteoblast differentiation is not well documented. In this study, we evaluated the effects of ERN on osteoclast and osteoblast differentiation in vitro.

**Results:**

ERN significantly reduced the formation of 1α,25(OH)_2_D_3_-induced tartrate-resistant acid phosphatase (TRAP)-positive cells at non-cytotoxic concentrations. Furthermore, ERN downregulated the mRNA expression of osteoclast-associated genes, such as nuclear factor of activated T cells cytoplasmic-1, TRAP, and cathepsin K. In addition, ERN suppressed mRNA expression of dendritic cell specific transmembrane protein (DC-STAMP), which encodes cell–cell fusion. However, ERN did not affect mineralization by osteoblasts. Thus, our data suggest that ERN may attenuate osteoclastic bone resorption by inhibiting multinucleation of mononuclear pre-osteoclasts and by suppressing mRNA expression of DC-STAMP in bone marrow cells without influencing mineralization by osteoblasts.

**Supplementary Information:**

The online version contains supplementary material available at 10.1186/s13104-022-05988-3.

## Introduction

Bone continuously repeats bone resorption and bone formation by osteoblasts [1]. Bone resorption and formation are stable under physiological conditions. However, when this balance is disturbed, bone structure and function become abnormal, resulting in various skeletal diseases, such as osteoporosis, rheumatoid arthritis, and periodontitis [2, 3]. In many cases, the activation of osteoclasts results in bone fragility. Therefore, it is important to identify the molecules that regulate osteoclast or osteoblast differentiation in maintaining bone homeostasis.

Osteoclasts are multinucleated cells that play key roles in mineralized bone matrix degradation. They are formed by fusing mononuclear precursors of the monocyte/macrophage lineage. On the other hand, osteoblasts are mononuclear cells, differentiate from mesenchymal cells, and are involved in the regulation of bone metabolism by synthesizing bone matrix, which becomes progressively mineralized. Osteoblasts are responsible for depositing hydroxyapatite and calcium phosphate crystals. Receptor activator of nuclear factor κ-B ligand (RANKL) produced by osteoblasts acts as an essential modulator of osteoclast differentiation and activation by directly binding to its receptor, RANK, which is expressed on osteoclast precursors and mature osteoclasts. RANKL specifically binds to its receptor, RANK, and regulates transcription factors such as c-Fos (a member of the dimeric transcription factor AP-1) and nuclear factor of activated T cells cytoplasmic-1 (NFATc1) [4]. In particular, NFATc1 are crucial activators of osteoclast-associated genes and activates target genes such as tartrate resistant acid phosphatase (TRAP) and cathepsin K (Ctsk) [5]. Then, osteoclast cells undergo fusion via cell–cell fusion molecules, such as dendritic cell-specific transmembrane protein (DC-STAMP) and osteoclast stimulatory transmembrane protein (OC-STAMP). These molecules are the main factors involved in the regulation of bone resorption, and DC-STAMP or OC-STAMP-deficient cells are not be able to develop into multinucleated osteoclasts [6, 7]. Importantly, osteoclast cell fusion by DC-STAMP and OC-STAMP activation is essential for the multinucleation of pre-osteoclasts.

**Fig. 1:**
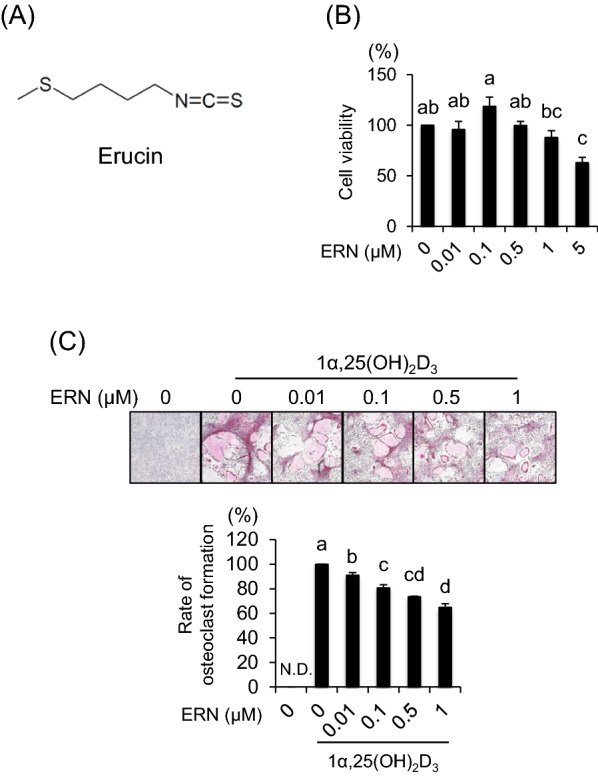
Effects of ERN on osteoclast formation in BMCs. **A** Chemical structure of ERN. **B** Effect of ERN on the cell viability of BMCs. The cytotoxic effect of ERN was evaluated using the CCK-8 assay. BMCs were treated with various concentrations of ERN (0–5 µM) for 6 days. Cell viability is expressed as a percentage of the values obtained for untreated ERN-cells. **C** BMCs were cultured with various concentrations of ERN (0–1 µM) in the presence of 1α,25(OH)_2_D_3_ (10^–8^ M) for 6 days. After incubation, the cells were fixed and stained for TRAP, a marker enzyme for osteoclast differentiation. TRAP-positive multinuclear cells (≥ 3 nuclei) were counted. The effect of ERN on osteoclast differentiation is expressed as the rate of multinucleated osteoclast formation, with ERN-untreated cells set at 100%. The data are expressed as the means ± SE of three independent experiments (n = 3). Means marked with different letters are significantly different (*P* < 0.05)

In cruciferous vegetables, many different glucosinolates yield isothiocyanate (ITC). Erucin (ERN) (Fig. 1A) is derived from arugula, a cruciferous vegetable, and induces apoptosis in several cancer cell lines [8, 9]. The anticancer activity of ITC is known to be mediated, at least in part, by induction of apoptosis and is associated with the presence of a –N = C = S moiety. Recently, we demonstrated that sulforaphane (SFN) and sulforaphene (SFE) inhibit osteoclast differentiation by suppressing the cell–cell fusion molecules DC-STAMP and OC-STAMP [10, 11]. SFN is the most extensively studied ITC in cruciferous vegetables, and ERN, being closely related to SFN, has also received attention because of its similar structure to SFN. However, since ERN does not contain oxidized sulfur, it might be expected that its biological effects would be weaker than those of SFN and SFE. Furthermore, the effects of ERN on bone metabolism have not yet been documented. In the present study, we investigated the effects of ERN on pre-osteoclast multinucleation and osteoblast differentiation in bone marrow cells (BMCs), which are similar to in vivo conditions.

## Main text

### Materials and methods

#### Materials

ERN was purchased from Cayman Chemical (Ann Arbor, MI, USA). 1α,25(OH)_2_D_3_ was obtained from Sigma-Aldrich (St. Louis, MO, USA). Osteoblast-inducer reagent was purchased from Takara Bio Inc. (Shiga, Japan). Soluble RANKL (sRANKL) was purchased from R&D Systems (Minneapolis, MN, USA). α-minimal essential medium (α-MEM) (phenol red-free) was obtained from Gibco BRL/Invitrogen (Carlsbad, CA, USA). Fetal bovine serum (FBS) was obtained from Biowest (Nuaillé, France). Cell Counting Kit-8 (CCK-8) was purchased from Dojindo (Kumamoto, Japan).

#### Cell culture

BMCs were obtained from two male ddY mice (8 week-old). The mice were purchased from Japan SLC Co. (Hamamatsu, Japan) and were fed the AIN-93G diet and given distilled water freely for three days during the acclimatization period. The mice were euthanized with an intraperitoneal injection of anesthesia (medetomidine hydrochloride 0.3 mg/kg + midazolam 4 mg/kg + butorphanol tartrate 5 mg/kg) followed by cervical dislocation. BMC samples were isolated from four femora and tibias, mixed, and collected by centrifugation at 6000 rpm for 20 s. in 2.0 mL microcentrifugation tubes, followed by α-MEM. BMC samples were randomly divided into two groups; a control group and an ERN treated group. The samples were used for each assay (each sample size (n): 3 or 6), i.e. cell viability (n = 3), TRAP stain (n = 3), mRNA expressions of osteoclastogenesis (n = 3), and Alizarin red stain (n = 6). The animal protocols and procedures used in this study were approved by the Tokyo University of Agriculture Animal Use Committee, and mice were maintained in accordance with the guidelines of the University for the care and use of laboratory animals. Marrow cells were flushed from bones, and cells were cultured in α-MEM (phenol red-free) supplemented with 10% FBS, 100 U/mL penicillin, and 100 μg/mL streptomycin (Gibco BRL/Invitrogen) at 37 °C in a humidified 5% CO_2_ atmosphere. RAW264.7 cells, mouse macrophage/monocytes, were obtained from the American Type Culture Collection (Manassas, VA, USA) and were cultured in α-MEM supplemented with 10% FBS, 100 U/mL penicillin, and 100 μg/mL streptomycin (Gibco BRL/Invitrogen) at 37 °C in a humidified 5% CO_2_ atmosphere.

#### Cytotoxicity assays

To evaluate the effect of ERN on the cell viability of BMCs, cytotoxicity assays were performed using the CCK-8. Briefly, BMCs (1 × 10^5^ cells/well) were cultured in 96-well plates. Then, treated with the presence or absence of ERN (0.01–5 µM) for 6 days in α-MEM containing 10% FBS. The effect of ERN on cell viability was calculated as percent cell viability, with ERN-untreated cells set at 100%.

#### Osteoclast differentiation assay

To form multinucleated osteoclasts, BMCs were differentiated into osteoclasts using 1α,25(OH)_2_D_3_. BMCs (1 × 10^6^ cells/well) were treated with 10^–8^ M of 1α,25(OH)_2_D_3_ to induce differentiation in the presence of ERN at a concentration of 0–1 µM in a 96-well plate for 6 days. After 6 days of incubation, the cells were fixed in 10% formaldehyde and then stained for TRAP, a marker enzyme of differentiated osteoclasts. TRAP-positive cells with ≥ 3 nuclei were scored as differentiated osteoclasts. The effect of ERN on osteoclast differentiation was calculated as the osteoclast formation rate, with ERN-untreated control cells set at 100%.

#### Real-time PCR analysis

BMCs (1 × 10^7^ cells/well) were seeded in a 24-well plate, treated with 10^–8^ M of 1α,25(OH)_2_D_3_ and various concentrations of ERN (0.1–1 μM) for 6 days. Total RNA was isolated from BMCs using Sepasol-RNA I Super G (Nacalai Tesque, Tokyo, Japan). Then, cDNA was synthesized from 500 ng of total RNA using reverse transcriptase (Takara Bio Inc.). Real-time PCR was performed using the ABI StepOnePlus System (Applied Biosystems, Foster City, CA, USA) and using 2 μL of the cDNA with THUNDERBIRD qPCR Mix (Toyobo, Osaka, Japan). The cycling conditions were 40 cycles of denaturation at 95 °C for 5 s and amplification at 60 °C for 30 s. The fold change compared to control was calculated according to the standard curve method. Real-time PCR was performed using the following primers: *c-Fos*, 5′-GAGTGATGCCGAAGGGATAA-3′ (forward) and 5′-GAGAAGCATTCCGGTCAGAG-3′ (reverse); *NFATc1*, 5′-GCTTCACCCATTTGCTCCAG-3′ (forward) and 5′-ATGGTGTGGAAATACGGTTGGTC-3′ (reverse); *TRAP*, 5′-ACTTCCCCAGCCCTTACTAC-3′ (forward) and 5′-TCAGCACATAGCCCACACCG-3′ (reverse); *Ctsk*, 5′-CCAGTGGGAGCTATGGAAGA-3′ (forward) and 5′-CTCCAGGTTATGGGCAGAGA-3′ (reverse); *DC-STAMP*, 5′-TCCTCCATGAACAAACAGTTCCA-3′ (forward) and 5′-AGACGTGGTTTAGGAATGCAGCTC-3′ (reverse); *OC-STAMP*, 5′-TGTCCTACAGTGCAGCCAAC-3′ (forward) and 5′-TCTCCTGAGTGATCGTGTGC-3′ (reverse); *β-Actin*, 5′-TGTCCACCTTCCAGCAGATGT-3′ (forward) and 5′-AGCTCAGTAACAGTCCGCCTAGA-3′ (reverse). All reactions were normalized to the housekeeping gene β-actin (*ACTB*).

#### Mineralization analysis

BMCs (1 × 10^6^ cells/well) were seeded in a 96-well plate for 24 h. Cells were then cultured with various concentrations of ERN (0–1 µM) in the presence of osteoblast-inducer reagents (ascorbic acid, β-glycerophosphate, and hydrocortisone) for 15 days. After incubation, the cells were fixed and stained with 1% alizarin red. For quantitative analysis, cells were destained with ethylpyridinium chloride and transferred to a 96-well plate to measure optical absorbance at 570 nm using a microplate reader. The effect of ERN on osteoblast differentiation is expressed as the degree of mineralization, with ERN-untreated cells set at 100%.

#### Statistical analysis

Results were presented as means ± SE of measurements performed on 3–6 cultures in each experimental or control group (there was no exclusion for any experimental unit.). All experiments were independently analyzed at least three times to confirm the results. For statistical significance, multiple comparisons were performed using Tukey’s test, after one-way analysis of variance (ANOVA). Statistical significance was set at *P* < 0.05.

### Results

#### Effects of ERN on osteoclast differentiation

We evaluated the cytotoxic effects of ERN in BMCs close to in vivo conditions using the CCK-8 assay. Low-dose ERN exerted no cytotoxicity, but decreased cell viability at a concentration of 5 µM (Fig. 1B). These results indicated that the maximum concentration of ERN used in our subsequent experiments (1 μM) had no cytotoxic effects toward BMCs. Next, to examine the effects of ERN on pre-osteoclast multinucleation, cells were incubated with ERN in the presence of 1α,25(OH)_2_D_3_ (Sigma-Aldrich). Compared to that reported for 1α,25(OH)_2_D_3_-treated cells, ERN decreased the rate of multinucleated osteoclast formation (Fig. 1C). It has been suggested that ERN inhibits pre-osteoclast multinucleation below cytotoxic concentrations.

#### Effects of ERN on the expression of osteoclast-associated genes

**Fig. 2:**
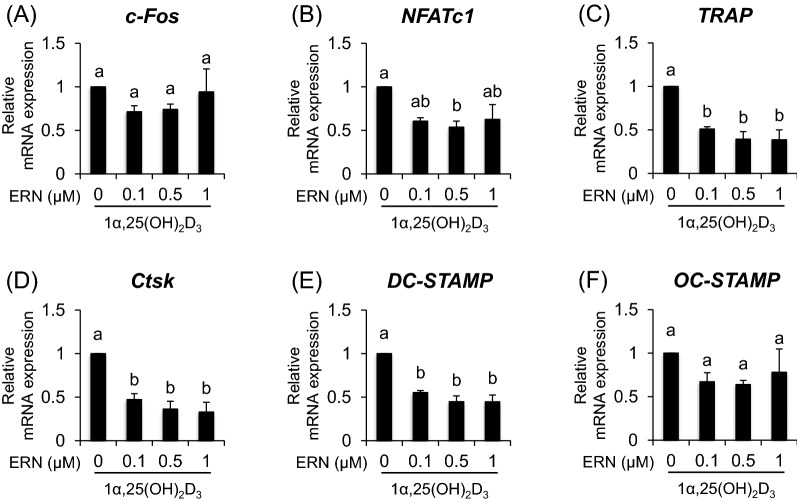
Effects of ERN on expression of osteoclast-differentiation associated genes. BMCs were cultured with various concentrations of ERN (0–1 µM) in the presence of 1α,25(OH)_2_D_3_ (10^–8^ M) for 6 days. mRNA expression levels of *c-Fos* (**A**), *NFATc1* (**B**), *TRAP* (**C**), *Ctsk* (**D**), *DC-STAMP* (**E**), and *OC-STAMP* (**F**) were analyzed by real-time PCR, and the results were normalized to the expression of the β-actin-encoding *ACTB* gene. The data are expressed as the means ± SE of three independent experiments (n = 3). Means marked with different letters are significantly different (*P* < 0.05)

We examined the effects of ERN on mRNA expression levels of osteoclast-associated genes such as *c-Fos*, *NFATc1*, *TRAP*, *Ctsk*, *DC-STAMP*, and *OC-STAMP*, using real-time PCR. Compared to that in 1α,25(OH)_2_D_3_-treated cells, ERN suppressed mRNA expression levels of *NFATc1*, *TRAP, Ctsk*, and *DC-STAMP* but not *c-Fos* and *OC-STAMP* (Fig. 2A–F).

#### Effects of ERN on osteoblast differentiation

**Fig. 3:**
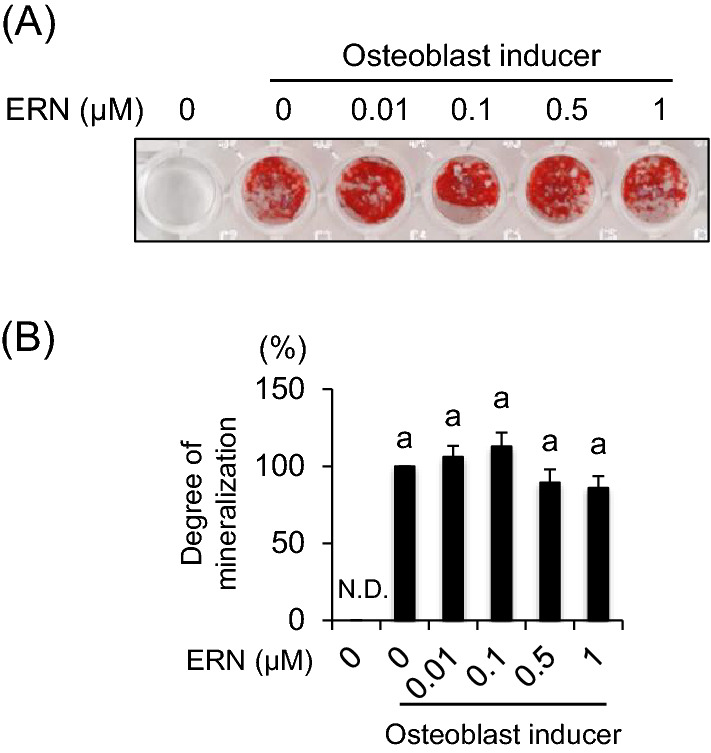
Effects of ERN on osteoblast differentiation in BMCs. **A** Mineralization of BMCs was assessed by alizarin red staining after 15 days of culture with osteoblast-inducer reagents (ascorbic acid, β-glycerophosphate, and hydrocortisone). **B** Staining activities were quantified by measure optical absorbance at 570 nm using a microplate reader. The effect of ERN on osteoblast differentiation is expressed as the degree of mineralization, with ERN-untreated cells set at 100%. The data are expressed as the means ± SE of multiple independent experiments (n = 6). Means marked with different letters are significantly different (*P* < 0.05)

To examine the effects of ERN in mineralization of osteoblast, BMCs were exposed to ERN during osteoblast formation. However, there was no difference in Alizarin red staining activities compared to ERN untreated cells (Fig. 3 A). In addition, similar results were obtained from the quantitative analysis of alizarin staining activity (Fig. 3 B).

### Discussion

In this study, ERN significantly inhibited osteoclast-differentiation and the expression of the osteoclast cell-fusion associated genes. Initially, we evaluated the effect of ERN on multinucleation of pre-osteoclasts using BMCs. As a result, ERN inhibited the multinucleation of pre-osteoclasts at non-cytotoxic concentrations. Furthermore, our results indicated that ERN suppresses the expression of the osteoclast differentiation-associated genes *NFATc1*, *TRAP*, *Ctsk*, and *DC-STAMP* but did not affect *c-Fos* and *OC-STAMP* expression. Multinucleated osteoclasts are derived from the fusion of monomeric osteoclasts, and fusion is considered an indispensable process for pre-osteoclast multinucleation and absorption of monomeric osteoclasts. Additionally, according to a previous study, targeted inhibition of DC-STAMP by siRNAs and specific antibody markedly suppressed the multinucleation of pre-osteoclasts [12]. Our data suggest that the inhibitory effect of ERN on the multinucleation of pre-osteoclasts can be attributed to the suppression of the cell–cell fusion molecule DC-STAMP. Additionally, the effects of ERN on cell viability and pre-osteoclast multinucleation of RAW 264.7, osteoclast precursor cells were similar to those observed in BMCs (Additional file 1: Fig. S1). On the other hand, we examined the effects of ERN on bone formation in primary osteoblast cultures. Mineralization nodules are biomarkers that determine osteoblast maturation and can be detected by Alizarin staining. When BMCs were cultured with osteoblast-inducer reagent, alizarin-stained mineralized bone nodules were detected on day 15. ERN did not inhibit the formation of mineralized bone nodules. These data suggest that ERN has very little effect on osteoblast mineralization, although ERN inhibits pre-osteoclast nucleation. Collectively, specific inhibition of osteoclast differentiation only may improve bone metabolism without affecting normal bone formation by osteoblasts. According to previous reports, many beneficial effects of ERN are due to its antioxidant and anti-cancer properties [13]. Osteoclasts produce reactive oxygen species (ROS). Free radicals are known to play important roles in osteoclast differentiation through activation of RANKL/RANK signaling [14]. Therefore, the antioxidant activity of ERN may be effective in inhibiting pre-osteoclast multinucleation. However, the inhibitory effect of ERN on pre-osteoclast multinucleation was weaker than that of SFN and SFE, as previously reported [10, 11]. Harris et al*.* [15] suggested that both SFN and ERN increase the expression of multidrug resistance protein 1 to a similar extent, although the effect of SFN was substantially greater than that of ERN. Furthermore, other studies have shown that ITCs with oxidized sulfur are the most efficient inducers of apoptosis and the biological activities of ITCs might be affected by the oxidation state of sulfur involving the side chains of such materials [16].

### Conclusion

In the present study, we demonstrated that ERN plays a novel role in inhibiting multinucleation of pre-osteoclasts by downregulating *DC-STAMP*. Notably, this is the first evidence that ERN inhibits multinucleation of pre-osteoclasts by suppressing cell–cell fusion without influencing mineralization in osteoblasts.

### Limitations

The precise mechanisms by which ERN improves bone metabolism are still unclear. In order to clarify the detailed molecular mechanism by which ERN suppresses osteoclast differentiation in vitro, it is necessary to study the molecules that control osteoclast fusion-related genes in addition to protein-level analysis. More bone resorption assay is also needed to clarify the inhibitory effect on osteoclastogenesis by ERN. Furthermore, in order to clarify the mechanism by which ERN improves bone metabolism in vivo, it is necessary to conduct experiments using animal models of osteoporosis.

## Supplementary Information

**Additional file 1: Fig. S1.** Effects of ERN on osteoclast differentiation in RAW 264.7 cells. (A) Cell viability of ERN-treated RAW 264.7. RAW 264.7 cells were cultured in a 96-well plate and then treated with various concentrations of ERN for 4 days. Cell viability was assessed using CCK-8 assays. Cell viability was analyzed and expressed as a percentage of the value of ERN-untreated cells. (B) RAW 264.7 cells were treated with various concentrations of ERN followed by sRANKL for 4 days. The cells were then stained with TRAP. TRAP-positive multinuclear cells (≥ 3 nuclei) were counted. The rate of osteoclast formation was analyzed and expressed as a percentage of the values of sRANKL-only treated cells (C–H). mRNA expression levels of *c-Fos*, *NFATc1*, *TRAP*, *Ctsk*, *DC-STAMP*, and *OC-STAMP* were analyzed by real-time PCR and the results were normalized to the expression of β-actin-encoding *ACTB*. The data are expressed as the means ± SE of three independent experiments (n = 3). Means marked with different letters are significantly different (*P* < 0.05).

## Data Availability

Raw data, including imaging files, and reagents described in this study will be made available upon request to the corresponding author, but some reagents, which we bought, should be made directly to the companies.

## Abbreviations

ERN: Erucin
SFN: Sulforaphane
SFE: Sulforaphene
ITC: Isothiocyanate
RANKL: Receptor activator of nuclear factor κ-B ligand
NFATc1: Nuclear factor of activated T cells cytoplasmic-1
TRAP: Tartrate-resistant acid phosphatase
Ctsk: Cathepsin K
DC-STAMP: Dendritic-cell specific transmembrane protein
OC-STAMP: Osteoclast stimulatory transmembrane protein
α-MEM: α-Minimal essential medium
CCK-8: Cell counting kit-8
BMCs: Bone marrow cells
