# Supplementary material for: Erucin inhibits osteoclast formation via suppressing cell–cell fusion molecule DC-STAMP without influencing mineralization by osteoblasts
Source: BMC Res Notes. 2022 Mar 16;15:105. doi: 10.1186/s13104-022-05988-3 (PMC8925049; doi:10.1186/s13104-022-05988-3)
Supplement: Supplementary file 1 — Additional file 1: Fig. S1. Effects of ERN on osteoclast differentiation in RAW 264.7 cells. (A) Cell viability of ERN-treated RAW 264.7. RAW 264.7 cells were cultured in a 96-well plate and then treated with various concentrations of ERN for 4 days. Cell viability was assessed using CCK-8 assays. Cell viability was analyzed and expressed as a percentage of the value of ERN-untreated cells. (B) RAW 264.7 cells were treated with various concentrations of ERN followed by sRANKL for 4 days. The cells were then stained with TRAP. TRAP-positive multinuclear cells (≥ 3 nuclei) were counted. The rate of osteoclast formation was analyzed and expressed as a percentage of the values of sRANKL-only treated cells (C–H). mRNA expression levels of c-Fos, NFATc1, TRAP, Ctsk, DC-STAMP, and OC-STAMP were analyzed by real-time PCR and the results were normalized to the expression of β-actin-encoding ACTB. The data are expressed as the means ± SE of three independent experiments (n = 3). Means marked with different letters are significantly different (P < 0.05). [file 13104_2022_5988_MOESM1_ESM.pptx]

## Slide 1
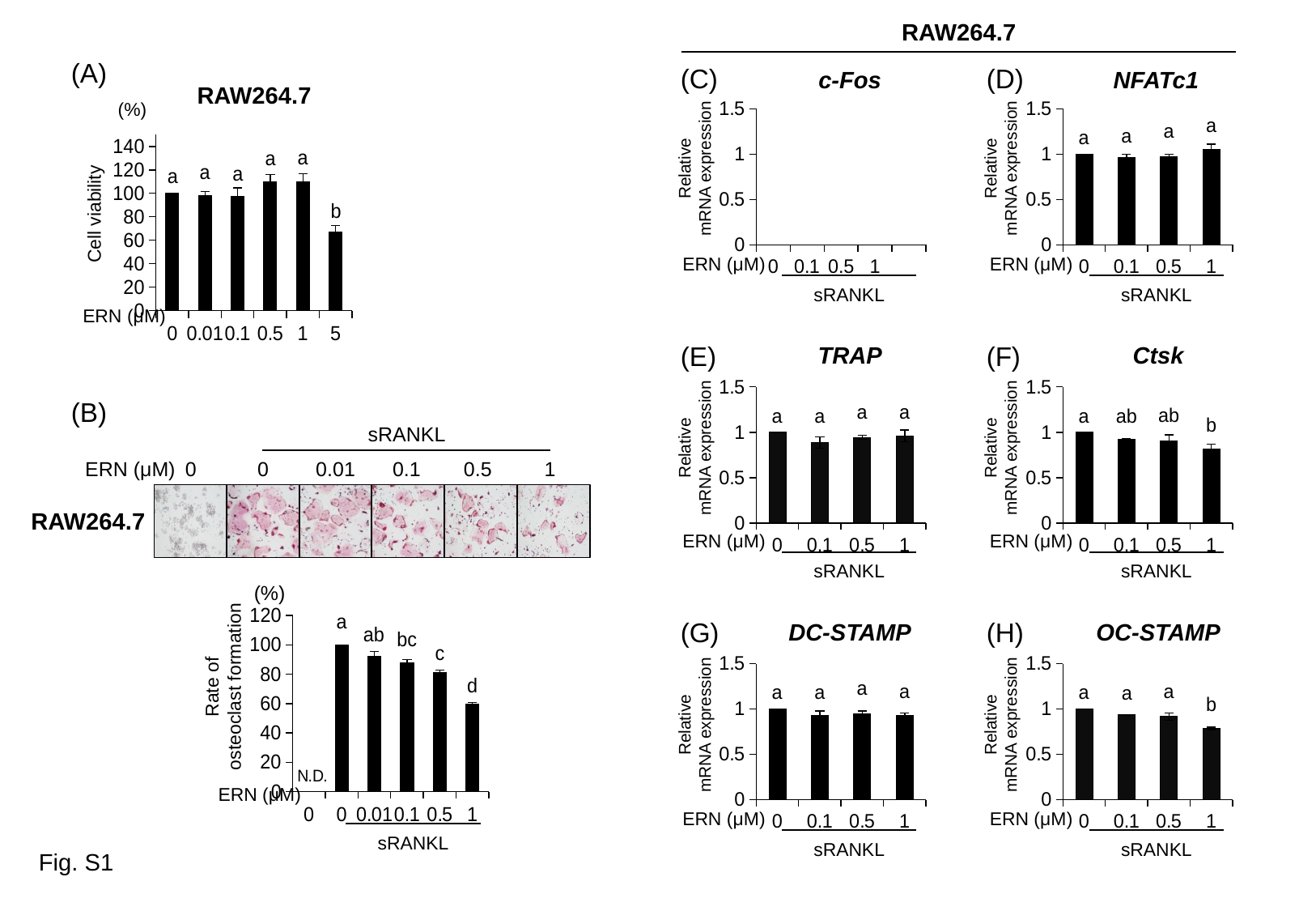

RAW264.7
(A)
(C)
(D)
c-Fos
NFATc1
RAW264.7
### Chart
| Category | |
|---|---|
| 0 | 1.0 |
| 0.1 | 0.8911489868740406 |
| 0.5 | 0.9986857386207193 |
| 1 | 1.0252332619309568 |
### Chart
| Category | |
|---|---|
| 0 | 1.0 |
| 0.1 | 0.963658816279089 |
| 0.5 | 0.9725508344155985 |
| 1 | 1.055256203484243 |(%)
### Chart
| Category | |
|---|---|
| 0 | 100.0 |
| 0.01 | 97.83891815687137 |
| 0.1 | 97.34528763280409 |
| 0.5 | 109.81070745569096 |
| 1 | 110.01860427346064 |
| 5 | 67.17006568076867 |Relative
mRNA expression
Relative
mRNA expression
Cell viability
ERN (μM)
sRANKL
ERN (μM)
sRANKL
ERN (μM)
(E)
(F)
TRAP
Ctsk
### Chart
| Category | |
|---|---|
| 0 | 1.0 |
| 0.1 | 0.8850253776620015 |
| 0.5 | 0.9436940826448706 |
| 1 | 0.9596102043108731 |
### Chart
| Category | |
|---|---|
| 0 | 1.0 |
| 0.1 | 0.9217584261214924 |
| 0.5 | 0.9083653789538456 |
| 1 | 0.8167209938334321 |(B)
sRANKL
ERN (μM)
0
0
0.01
0.1
0.5
1
Relative
mRNA expression
Relative
mRNA expression
RAW264.7
ERN (μM)
sRANKL
ERN (μM)
sRANKL
(%)
### Chart
| Category | |
|---|---|
| 0 | 0.0 |
| 0 | 100.0 |
| 0.01 | 92.41645244215938 |
| 0.1 | 87.78920308483292 |
| 0.5 | 80.97686375321337 |
| 1 | 59.38303341902314 |(G)
(H)
DC-STAMP
OC-STAMP
### Chart
| Category | |
|---|---|
| 0 | 1.0 |
| 0.1 | 0.9308157989526077 |
| 0.5 | 0.9505881386346684 |
| 1 | 0.9256214643754285 |
### Chart
| Category | |
|---|---|
| 0 | 1.0 |
| 0.1 | 0.9337990102837644 |
| 0.5 | 0.9174327544786646 |
| 1 | 0.7841734411205817 |Rate of
osteoclast formation
Relative
mRNA expression
Relative
mRNA expression
ERN (μM)
sRANKL
ERN (μM)
sRANKL
ERN (μM)
sRANKL
Fig. S1
